# Genome-wide detection and sequence conservation analysis of long non-coding RNA during hair follicle cycle of yak

**DOI:** 10.1186/s12864-020-07082-z

**Published:** 2020-10-01

**Authors:** Xiaolan Zhang, Qi Bao, Congjun Jia, Chen Li, Yongfang Chang, Xiaoyun Wu, Chunnian Liang, Pengjia Bao, Ping Yan

**Affiliations:** Key Laboratory of Yak Breeding Engineering Gansu Province, Lanzhou Institute of Husbandry and Pharmaceutical Sciences, Chinese Academy of Agricultural Sciences, Lanzhou, 730050 China

**Keywords:** Hair follicle cycling, lncRNA, NCBI blast-2.9.0 +, Yak

## Abstract

**Background:**

Long non-coding RNA (lncRNA) as an important regulator has been demonstrated playing an indispensable role in the biological process of hair follicles (HFs) growth. However, their function and expression profile in the HFs cycle of yak are yet unknown. Only a few functional lncRNAs have been identified, partly due to the low sequence conservation and lack of identified conserved properties in lncRNAs. Here, lncRNA-seq was employed to detect the expression profile of lncRNAs during the HFs cycle of yak, and the sequence conservation of two datasets between yak and cashmere goat during the HFs cycle was analyzed.

**Results:**

A total of 2884 lncRNAs were identified in 5 phases (Jan., Mar., Jun., Aug., and Oct.) during the HFs cycle of yak. Then, differential expression analysis between 3 phases (Jan., Mar., and Oct.) was performed, revealing that 198 differentially expressed lncRNAs (DELs) were obtained in the Oct.-vs-Jan. group, 280 DELs were obtained in the Jan.-vs-Mar. group, and 340 DELs were obtained in the Mar.-vs-Oct. group. Subsequently, the nearest genes of lncRNAs were searched as the potential target genes and used to explore the function of DELs by GO and KEGG enrichment analysis. Several critical pathways involved in HFs development such as Wnt signaling pathway, VEGF signaling pathway, and signaling pathways regulating pluripotency of stem cells, were enriched. To further screen key lncRNAs influencing the HFs cycle, 24 DELs with differ degree of sequence conservation were obtained via a comparative analysis of partial DELs with previously published lncRNA-seq data of cashmere goat in the HFs cycle using NCBI BLAST-2.9.0+, and 3 DELs of them were randomly selected for further detailed analysis of the sequence conservation properties.

**Conclusions:**

This study revealed the expression pattern and potential function of lncRNAs during HFs cycle of yak, which would expand the knowledge about the role of lncRNAs in the HFs cycle. The findings related to sequence conservation properties of lncRNAs in the HFs cycle between the two species may provide valuable insights into the study of lncRNA functionality and mechanism.

## Background

Yak (*Bos grunniens*) is the only domesticated breed in the *Bos* genus that can produce wool. It has been regarded as “all-purpose” livestock to provide essential living and economy resource for inhabitants around the Qinghai-Tibetan Plateau. The hair growth cycle of yak is similar to that of cashmere goat, also presenting a seasonal pattern under the control of the animals’ endocrine systems and photoperiod, including anagen (growth), catagen (regression), and telogen (rest) [1, 2]. The morphological changes in hair growth take place following different phases of the HF cycle [3]. In the anagen phase, an entire hair shaft from follicles is produced, this phase determines the length of the hair shaft. The catagen phase is the dynamic transition from the anagen phase to the telogen, in which the HFs regress in a lower cell cycling process caused by the increased apoptosis of epithelial cells in the bulb, outer root sheath, and outermost epithelial layer. During the telogen phase, follicles regenerate with the activation of multipotent stem cells, receiving signals to initiate a new cycle of hair growth [4]. The transition between the phases is the result of multiple molecular signals and their intricate interactions in the skin during HF development. Several signaling pathways, such as Wnt, Notch, Hedgehog, and bone morphogenetic protein (BMP), are crucial for the regulation of HFs cycle [5]. Moreover, some hormones and molecules, such as melatonin, estrogen, fibroblast growth factor 5 (*FGF5*), vascular endothelial growth factor (*VEGF*), and so on, have been identified influencing the development of the HFs cycle [6–9].

An increasing number of studies have shown that long non-coding RNAs (lncRNAs) play vital roles in regulating various biological processes, such as development, cell proliferation, and differentiation [10, 11]. LncRNAs regulate gene expression at various genomic levels, including chromatin modification, transcription, and post-transcriptional regulation, and multiple binding modules are involved that affect the gene expression in the immediate genomic vicinity (in *cis*) or at other genomic locations (in *trans*) [12–14]. LncRNAs are crucial for maintaining pluripotency and lineage commitment [15]. In dermal papilla, lncRNAs regulate the gene expression associated with HFs development and postnatal hair cycling [16]. In addition, lncRNAs have been found to participate in the regulation of the HFs cycle in cashmere goats, sheep and Angora rabbit, besides regulating skin pigmentation in goats and cattle [17–20]. So far, the studies on HF growth in yak focused mainly on the expression pattern of several genes such as *BMP2*, *TGF-β* and *HSP70* [21–23]. No systematic molecular study reported on the HFs cycle in yak. The hair growth pattern of yak is similar to that of goats. Also, the harsh living environment with cold and oxygen-thin air gives yak some unique traits, such as the high altitude adaptation [24]. The skin is an important protective barrier against a cold and harsh environment. Studies on the influence of lncRNAs on HFs may broaden the knowledge on the alpine adaptation of yak.

The functions of lncRNAs were predicted by analyzing the role of their potential targets in *cis* or *trans* co-localization or correlation. Only a small fraction of lncRNAs have been experimentally tested for their function [12, 25], including the well-studied lncRNAs Xist, HOTAIR, and MALAT1 that were synchronously reported to have higher sequence conversation or a conservative domain [26–28]. In fact, contrary to coding genes, lncRNAs were mostly reported with a lower level of sequence conservation and lacked identified conservation properties across species [12], hindering the studies on lncRNAs function. The findings about lncRNAs in position and evolution conservation are helpful to further understand their roles in various biological processes. LncRNAs were found to be ancient components in the evolution of vertebrate genome and showed an unprecedented evolutionary plasticity, and the lncRNAs conserved in position were primarily connected to many developmental transcription factors [29, 30]. These findings indicated the importance of lncRNAs in genome evolution and development from the view of functional commonality. However, the functional exploration of individual lncRNAs needed to be performed with elaborate analysis. Recent studies demonstrated the secondary structure conservation of homologous lncRNAs among species [13], highlighting the significance of structure in functionality of lncRNAs, along with species-specific and spatiotemporal expression patterns [31]. The sequence conservation of lncRNAs for the same trait at the interspecies level is worth exploring. The growth pattern of HFs in yak and cashmere goat provided a good animal model for the sequence conservation study of lncRNAs in the same trait.

The objective of this study was to investigate the expression pattern of lncRNAs during HFs cycle of yak and detect the potential function of lncRNAs involved. The sequence conservation of partial lncRNAs between yak and cashmere goat during HFs cycle was analyzed to obtain more critical lncRNAs associated with the HFs cycle of yak. The present study provided a systematic knowledge about lncRNAs regulating the HFs cycle of yak and screened lncRNAs that were sequence-conserved between yak and goat, thus providing insights into the role of lncRNAs in hair growth biology and laying a foundation for further functional studies.

## Methods

### Animals and sample collection

Tianzhu white yaks with fine fiber production trait were used in this study. All the yaks were obtained from the Tianzhu white yak propagation bases of Gansu province, China. Female yaks about 2 years old in similar shape (coefficient of relationship < 0.125) were selected for sample collection in different phases of the HFs cycle that included the following five time points: Jan., Mar., Jun., Aug., and Oct.. Skin samples of 3 yaks at each point were collected from the scapulae region using a medical skin sampler after local injection of 2% lidocaine and rapidly frozen in liquid nitrogen for further processing. Veterinary streptomycin sulfate and penicillin potassium powders were applied to the wound and put a sterile dressing over the wound. The animal wounds can heal completely within a month. The samples were collected at the adjacent site in each distinct phase.

All the experimental procedures involved in this study were approved by the Animal Management and Ethics Committee of the Lanzhou Institute of Animal Science and Veterinary Medicine, Chinese Academy of Agricultural Sciences (Permit No. SYXK-2016-0039).

### RNA isolation, library preparation, and sequencing

Total RNA of skin tissues was extracted using TRIzol reagent (Invitrogen, CA, USA) following the manufacturer’s protocols, after grinding them in liquid nitrogen. RNA integrity was analyzed using an RNA Nano 6000 Assay Kit of the Bioanalyzer 2100 system (Agilent Technologies, CA, USA). RNA concentration and purity were measured with the Nanodrop 2000 photometer spectrophotometer (Implen, CA, USA). Approximately 3 μg RNA was used for RNA library preparation. First, ribosomal RNA was removed using TruSeq Stranded Total RNA with a Ribo-Zero Gold Kit (Illumina, CA, USA). Subsequently, interruption reagent was added to break the rRNA-depleted RNA into short fragments, and the latter RNA was used as a template. Random hexamer primers were used to synthesize the first-strand cDNA, and then the second-strand cDNA was synthesized using DNA polymerase I and RNase H. In the reaction system of second-strand cDNA, dUTP was used instead of dTTP. Then, different adapters were connected, and the UNG enzyme method was used to digest the strand containing dUTP, only retaining the first cDNA strands with different linkers. After purification of the cDNA strands, the remaining overhangs were converted into blunt ends via polymerase/exonuclease activities, and adenylation of the 3′ ends were performed. The sequencing adapter was ligated and the cDNA fragments of 150–200 bp in length were selected to perform PCR amplification. Finally, the PCR products were purified (AMPure XP system), and RNA library quality was assessed with an Agilent 2100 Bioanalyzer system (Kapa BioSystems, MA, USA). The clustering of the index-coded samples was performed using TruSeq PE Cluster reagent (Illumina, CA, USA) on a cBot Cluster generation system following the manufacturer’s protocols. Then, the cDNA libraries were subjected to standard paired-end sequencing with an Illumina Hiseq 2500 platform and 150 bp paired-end reads were generated.

### Quality control and transcriptome assembly

Raw reads were trimmed by removing adapter sequences, reads containing over 10% of poly -N, and low-quality bases (> 50% of bases whose Phred scores were < 5%) using in-house Perl scripts. The valid bases, Phred score (Q30) and GC content were used to filter high quality clean data. The alignment of paired-end clean reads to the reference genome (versionGCA_005887515.2 BosGru3.0) was performed using the hisat2 [32]. The mapped reads of each library were assembled using Cufflinks (v2.1.1) in a reference-based approach [33]. For the prediction of lncRNAs, reassembling of transcripts was performed using a streaming neural network algorithm with StringTie software [34], which could accurately determine the direction of transcript chains for the data of chain-specific library.

### Identification of lncRNAs

Based on the characteristics of lncRNAs, a strict four-step procedure was used to identify lncRNAs from the assembled transcripts: (1) spliced transcripts were compared with reference transcripts using Cuff-compare v2.1.1 [33] to remove known coding transcripts or loci, and the location type of the remaining transcripts was determined; (2) transcripts with length < 200 bp or exon number < 2 were removed; (3) the coding potential of transcripts was analyzed to remove transcripts with coding potential. The analytic software was CNCI (v2) [35], CPC (0.9-r2) [36], Pfam-scan (v1.3) [37], and PLEK [38]. (4) LncRNA transcripts obtained in the third step were used to align the annotated lncRNAs in yak with Blastn software, the transcripts overlapped with known lncRNAs of yak were removed, and novel lncRNAs were screened. Then, novel lncRNAs and known lncRNA transcripts were used for further quantitative analysis. FEELnc [39] was used to classify and count the different types of identified novel lncRNAs based on the positional relationship with their closest protein coding transcripts; the classification involved four sides including direction (sense or antisense), type (genic or intergenic), location (e.g. upstream for lincRNA or exonic for genic lncRNAs), and subtype (e.g. divergent for lincRNAs or containing for genic lncRNAs).

### Differential expression analysis of lncRNA

Fragments per kilobase for a million reads (FPKM) of lncRNA transcripts was calculated with Cuffdiff (v2.1.1) [40]**.** DESeq [41] was used to normalize the lncRNA counts of each skin sample (basemen value to estimate the expression). *P* value < 0.05 and foldchange > 2 was set as the threshold for significantly differential expression between any two distinct phases of HFs growth. Based on the count data of lncRNA expression, sample similarities of the three groups (Jan., Mar., and Oct.) was analyzed via principal component analysis (PCA). Besides, heatmap showing the Euclidean distance between the samples as calculated from the variance stabilizing transformation of the count data, also was used to check the consistency of the biological replicates.

### Function enrichment analysis of the nearby genes of differential lncRNAs

The nearest protein-coding gene paired with each lncRNA was searched as the potential regulatory target gene of that lncRNA. If no gene was detected within 100 kb upstream or downstream of a lncRNA, that lncRNA was excluded in subsequent analysis. Function enrichment analysis of the target genes between any two groups DELs were performed, using the clusterProfiler R package depends on gene ontology (GO) database and the Kyoto Encyclopedia of Genes and Genomes (KEGG) database, to calculate enrichment test for GO terms and KEGG pathways based on hypergeometric distribution [42]. The protein-protein interaction network of all the nearby genes paired with DELs was analyzed with the STRING database (https://string-db.org/), and further visualized with Cytoscape V3.5.1 (http://www.cytoscape.org/).

### Verification of sequencing data using real-time quantitative PCR

Total RNA from the skin tissue subjected to RNA sequencing was also used to verify the sequencing results by real-time quantitative PCR (RT-qPCR). The first-strand cDNA was obtained using a PrimeScript RT reagent kit with gDNA Eraser (TaKaRa, Dalian, China). The RT-qPCR was performed using SYBR Premix Ex Taq II (TaKaRa) on the Bio-Rad CFX96 TouchReal-Time PCR Detection System (Bio-Rad, CA, USA). The reaction conditions were set as follows: 95 °C for 3 min, followed by 40 cycles of 95 °C for 12 s and 60 °C for 30 s. The relative expression of lncRNAs and mRNAs was analyzed with the 2^−ΔΔCt^ method and normalized using *GAPDH*. The primers used for RT-qPCR were designed with oligo 6 and are listed in Additional file 1.

### Sequence conservation analysis of lncRNAs between yak and cashmere goat during HFs cycle

To further screen the key lncRNAs in the HFs cycle of yak, DELs paired with differently expressed genes (DEGs) were screened by comparing analysis the lncRNA data with our previous mRNA data [43]. Then, these DELs were used for sequence conservation analysis with the lncRNA data of cashmere goat during the HFs cycle using NCBI BLAST-2.9.0+ [44] with parameters of“Gap Penalties: Existence 0, Extension 2.5, Expectation value 1e-10”. The lncRNA dataset of the cashmere goat was used as “blastdb database”, and the DELs of the yak were used as “query database”. The lncRNA dataset of the cashmere goat was downloaded from a previous study [19]. The sequence conservation properties were analyzed using dottup website, WebLogo3 (http://weblogo.threeplusone.com/), Clone Manager bioinformatics software, and so forth.

## Results

### Identification and differential expression analysis of lncRNAs in the yak skin

**Fig. 1:**
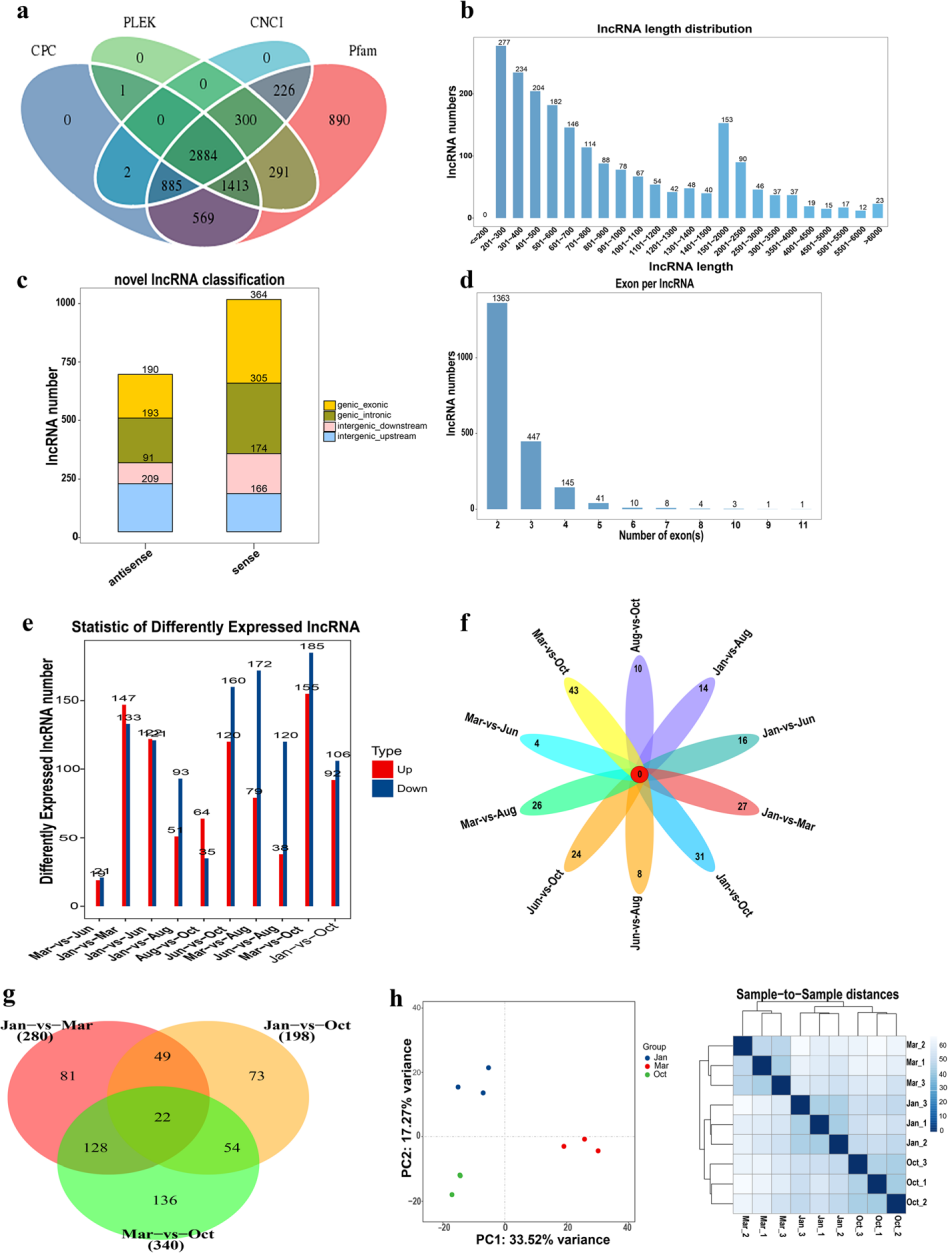
Long noncoding RNAs (lncRNAs) identification and comparative analysis of their differential expression in the hair follicles (HFs) cycle of yak. **a** Screening of the candidate lncRNAs in skin transcriptome. Four tools (CPC, PLEK, CNCI, and PFAM) were employed to analyze the coding potential of lncRNAs. Those lncRNAs shared by the four analytical tools were designated as candidate lncRNAs and used for subsequent analysis. **b** The length distribution, **c** classification, and **d** exon number distribution of novel lncRNAs. **e** Statistical analysis of differently expressed lncRNAs in every two comparison groups. **f** The common and specific differently expressed lncRNAs between different comparison groups (right). **g** Venn diagrams of DELs in three comparison groups, including Jan.-vs-Mar., Jan.-vs-Oct. and Mar.-vs-Oct., the lncRNAs shared with the three groups indicated differently expressed in every comparison group. **h** PCA and the analysis of sample-to-sample distance were used to check the sample similarities of the three groups (Jan., Mar. and Oct.)

A total of 748.61 M raw reads were produced from 15 skin samples in 5 phase groups of yaks using the Illumina Hiseq 2500 platform, in which 733.79 M reads were clean (Additional file 2), accounting for 98% of raw reads. After screening with rigorous criteria and analyzing the coding potential with the software CNCI, CPC, Pfam and PLEK, 2884 lncRNAs from all samples were identified (Fig. 1a). Of these transcripts, 70.1% (2023) were novel lncRNAs. The average length of novel lncRNAs was 1152 bp (Fig. 1b). Furthermore, the data showed that approximately 40% of the novel lncRNAs were antisense lncRNAs and 60% were sense lncRNAs (Fig. 1c), besides approximately 90% of the novel lncRNAs with two to three exons (Fig. 1d). These data of lncRNAs were similar to the features of previously described lncRNAs [45].

The expression levels of lncRNAs were analyzed using Cuffdiff v2.1.1. The DELs of every two distinct groups were screened with the threshold set as fold change > 2 and *p* value < 0.05 (Additional file 3). Numbers of all the DELs between every comparison groups, and the common and specific DELs were visualized and were shown in Fig. 1e and f. Based on the result, Oct. was selected in the anagen phase with Jan. (catagen) and Mar. (telogen) for further functional analysis, because both Mar.-vs-Oct. and Jan.-vs-Oct. comparison groups had a higher number of DELs, while the number of DELs was relatively minor in the Mar.-vs-Jun. and Aug.-vs-Oct. comparison groups. Jun. and Aug. were preliminarily excluded to simplify subsequent analysis. In the differential expression analysis of the 3 groups (Jan., Mar. and Oct.), 280 DELs were obtained in the Jan.-vs-Mar., 340 and 198 DELs were obtained in the Mar.-vs-Oct. and Jan.-vs-Oct. comparison groups, respectively. Also, 22 of these DELs were shared in the 3 comparison groups (Fig. 1g). PCA was used to analyze the sample similarities. A more concentrated distribution of samples in the same group was clearly observed. In addition, Fig. 1h showed that the sample-to-sample distances are closer in the same group. The data indicated that the samples had high reliability.

### GO and KEGG enrichment analysis

**Fig. 2:**
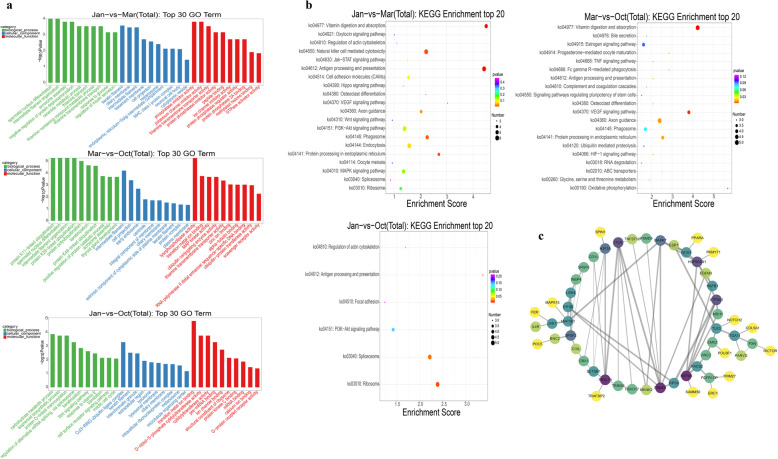
GO and KEGG enrichment analysis of the nearest genes of differently expressed lncRNAs. **a** GO analysis of the nearest genes of differentially expressed lncRNAs; the top 30 GO terms (top 10 terms in each GO category) are shown. **b** KEGG enrichment analysis of the nearest genes of differently expressed lncRNAs; the top 20 pathways with the number of genes greater than two are illustrated as bubble plot. The size of the bubble indicates gene number, and the color indicates *P* value. **c** Protein-to-protein interactive network of all the nearest genes of differently expressed lncRNAs visualized using Cytoscape. The color indicates node degree

The nearest protein-coding genes paired with DELs were used to perform GO and KEGG enrichment analysis so as to investigate the potential function of DELs during the three distinct HFs phases. Figure 2a shows the top 30 GO terms (top 10 terms in each GO category). The result showed that the GO terms were enriched mainly in Keratinization, keratin filament, carbohydrate metabolic process, and thiamine transport, which are involved in hair formation or nutrient metabolism. In the Mar.-vs-Oct. comparison group, protein ubiquitination was highly enriched. The ubiquitin-mediated proteolysis pathway was reported to be important for distinguishing the secondary hair follicles (SFs) from primary hair follicles (PFs) in cashmere goats [46, 47], suggesting that the regulation of protein ubiquitination was related to the transition between PFs and SFs from Mar. to Oct. in the present study.

KEGG enrichment analysis was performed. The top 20 KEGG pathways with the number of DELs greater than 2 are shown in Fig. 2b. The VEGF signaling pathway, Wnt signaling pathway, and signaling pathways regulating the pluripotency of stem cells, which were reported to play indispensable roles during the HFs cycle, were enriched [48–50]. In addition, hormone and nutrition metabolism signaling pathway (e.g. estrogen signaling pathway, vitamin digestion and absorption, and glycine, serine, and threonine metabolism), immune response signaling pathway (e.g. antigen processing and presentation and natural killer cell–mediated cytotoxicity) were enriched, which might be essential for the HFs growth [6, 51]. The pathways among distinct phases coincidentally corresponding with the biological process of hair cycling seemed to orchestrate a dynamic genesis of the HFs cycle at the molecular level. For example, in Jan.-vs-Mar. comparison group, the only up-regulated pathway was vitamin digestion and absorption due to the relative resting state in the telogen (Mar.) and reserve energy for the next HFs cycle (Figure S1). Moreover, the HIF-1 signaling pathway involved in the perception of hypoxia was enriched, which might be related to the high-altitude environment with frigid and hypoxia of yak living. The protein-protein interaction networks of coding genes that paired with all the DELs of the three distinct phases were analyzed using the STRING database (https://string-db.org). The result showed that *ITCH*, *PSMD1*, *FBXL19*, and *HSPA9* primarily involved in protein ubiquitination (queried from the UniProt database) were key nodes of the DELs paired genes (Fig. 2c). The ubiquitin-mediated proteolysis pathway was important for distinguishing SFs from PFs in cashmere goats [46, 47], and it was recently reported that the ubiquitylation process associated with Hedgehog signaling a crucial signaling that affecting on HFs regeneration [52–54].

### Comparative analysis of DELs in yak with cashmere goat lncRNA data

**Fig. 3:**
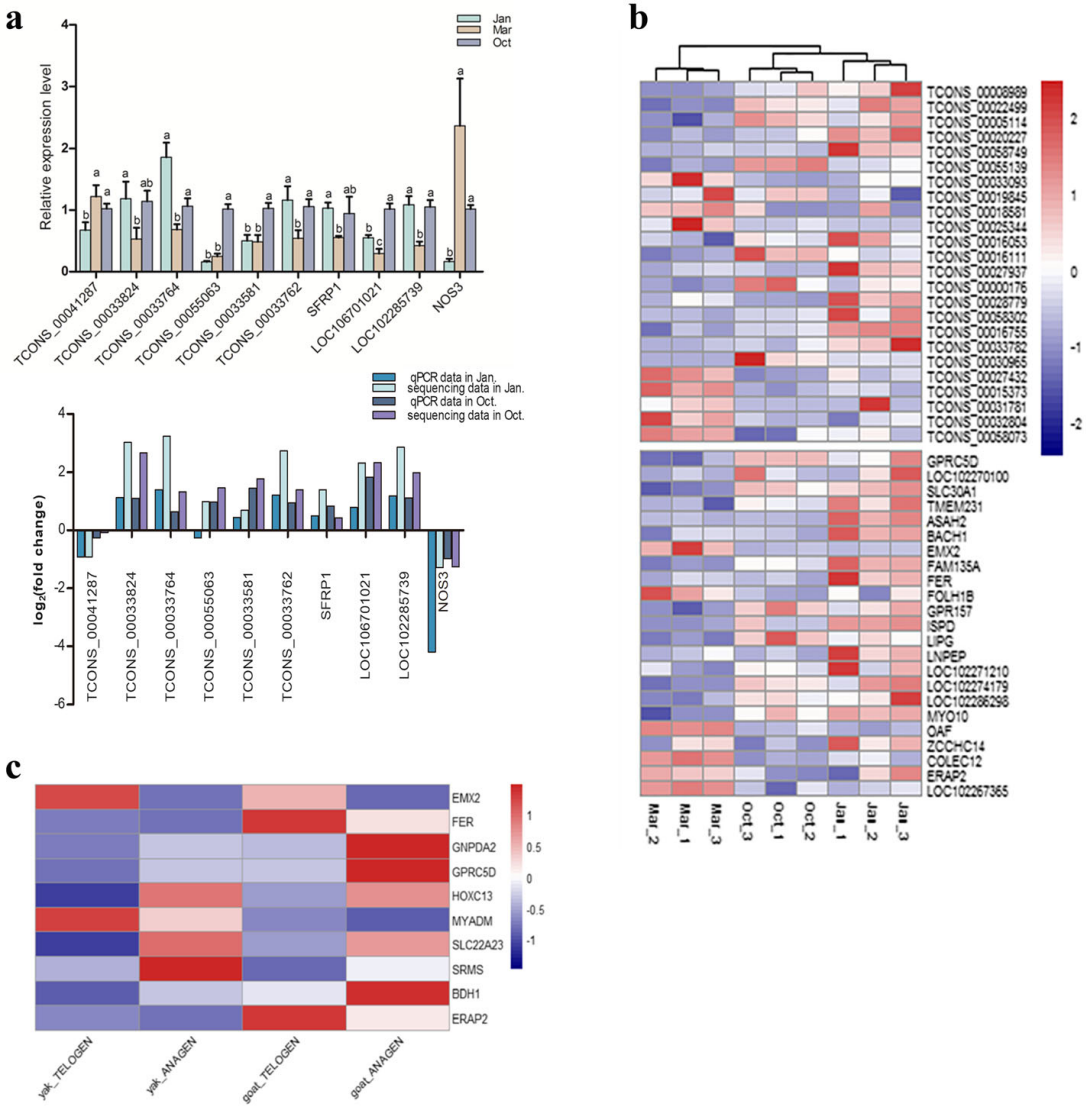
Verification of sequencing data by RT-qPCR and screening of sequence conserved DELs between yak and cashmere goat. **a** The expression level of differently expressed lncRNAs and several paired DEGs were detected by RT-qPCR (above), the relative expression levels of lncRNAs and mRNAs were analyzed by the 2^−ΔΔCt^ method and normalized using *GAPDH*. Data were presented as means ± SEM (*n* = 3). The same letter means no significant difference, different letters mean significant difference; Comparation of the expression pattern of the sequencing data and RT-qPCR data (below). Log2(fold change) > 0 indicates the transcript up regulated in Jan. (catagen) or Oct. (anagen) compared to Mar.(telogen). Log_2_(fold change) < 0 is the opposite. **b** Cluster analysis of the aligned 24 DELs and their paired 23 differently expressed mRNAs in yak. **c** Heat map showing the expression profiles of DEGs in both yak and cashmere goat during the HFs cycle

To further screen the DELs that are more likely to affect the HFs cycle in yak, 93 differently expressed protein coding genes (DEGs) paired with 110 DELs were obtained by combining the present data with our previous mRNA data on the HFs cycle in yak [43]. The detailed information on these DELs and DEGs were presented in Additional file 4. The expression tendency and abundance of most DELs were consistent with the paired coding genes, although a few pairs such as TCONS_00055063 with its paired gene NOS3 had an opposite expression tendency. Six lncRNAs and four lncRNA-paired protein-coding genes, which were differentially expressed in at least any two distinct phases, were selected to verify the transcriptome sequencing data by RT-qPCR. LncRNAs were selected based on the expression levels and potential function of the paired protein-coding gene in hair development. Figure 3a shows the RT-qPCR result and the comparison of the sequencing data and RT-qPCR data.

LncRNAs have species and tissue specificity with lower sequence conservation [11]. Yak and cashmere goat belong to the Bovidae family of ruminants, and they had a similar seasonal HFs cycle. To explore the sequence conservation level between yak and cashmere goat during the HFs cycle and obtain more reliable DELs regulating HFs growth, NCBI BLAST-2.9.0+ was used to detect the sequence-conserved lncRNAs of these 110 DELs with the lncRNA data of the cashmere goat in the HFs cycle (532 lncRNA sequences) [19]. Sequences information of all the lncRNAs of yak in this study was showed in Additional file 5. The BLAST result found that 24 DELs among these 110 DELs of yak had sequence identities with cashmere goat lncRNA data to different degrees. Then, the expression profiles of aligned DELs in the yak with their respective alignments in the cashmere goat dataset were comparatively analyzed. Excluding the repetitive lncRNA sequences, a total of 287 alignments including 125 lncRNA sequences in the cashmere goat dataset were aligned by 24 DELs of the yak dataset (Additional files 6 and 7). The aligned lncRNA sequences in both datasets all accounted for approximately 20% (125/532, 24/110) of their total databases used in BLAST-2.9.0+ (Additional file 6). Among the 125 lncRNA sequences, 41 were found to be differentially expressed in the cashmere goat during the HFs cycle [19]. Comparative analysis found that the expression profiles of most of the differently expressed alignments (approximately 80%) in the cashmere goat in anagen and telogen were similar to those of the aligned DELs in the yak in anagen (Oct.) and the transition from catagen (Jan.) to telogen (Mar.) (Additional file 8). This finding suggested that the number of lncRNAs with a conserved sequence is limited, accounting for about 20%. The expression pattern of the sequence conserved DELs between the yak and cashmere goat data had a little difference, which probably related to the difference from species and sampling time, considering only two phases of the HFs cycle in the cashmere goat. Or not all kinds of conserved elements in the lncRNAs play the similar roles between species. Further experiments are needed to detect whether the DELs with conserved elements between the yak and cashmere goat playing similar functions during the HFs cycle.

Figure 3b shows the 24 DELs and their paired 23 DEGs of the yak with a heat map. In addition, comparative analysis of the 93 DEGs in the yak with the DEGs between the anagen and telogen of cashmere goat data [19] found that 10 DEGs were differentially expressed both in the yak and cashmere goat during the HFs cycle and the expression tendency in the cyclic process was consistent (Fig. 3c). The paired lncRNAs of genes *GPRC5D*, *FER*, *EMX2* and *ERAP2* simultaneously had a conserved sequence between the yak and cashmere goat. *GPRC5D* and *EMX2* were associated with HFs morphogenesis [55, 56]. These results verified the sequencing data and further revealed some potentially important DELs and their paired genes affecting the HFs cycle based on the comparative analysis of lncRNA-seq data between the yak and cashmere goat.

### Sequence conservation properties analysis of lncRNAs between yak and cashmere goat

**Fig. 4:**
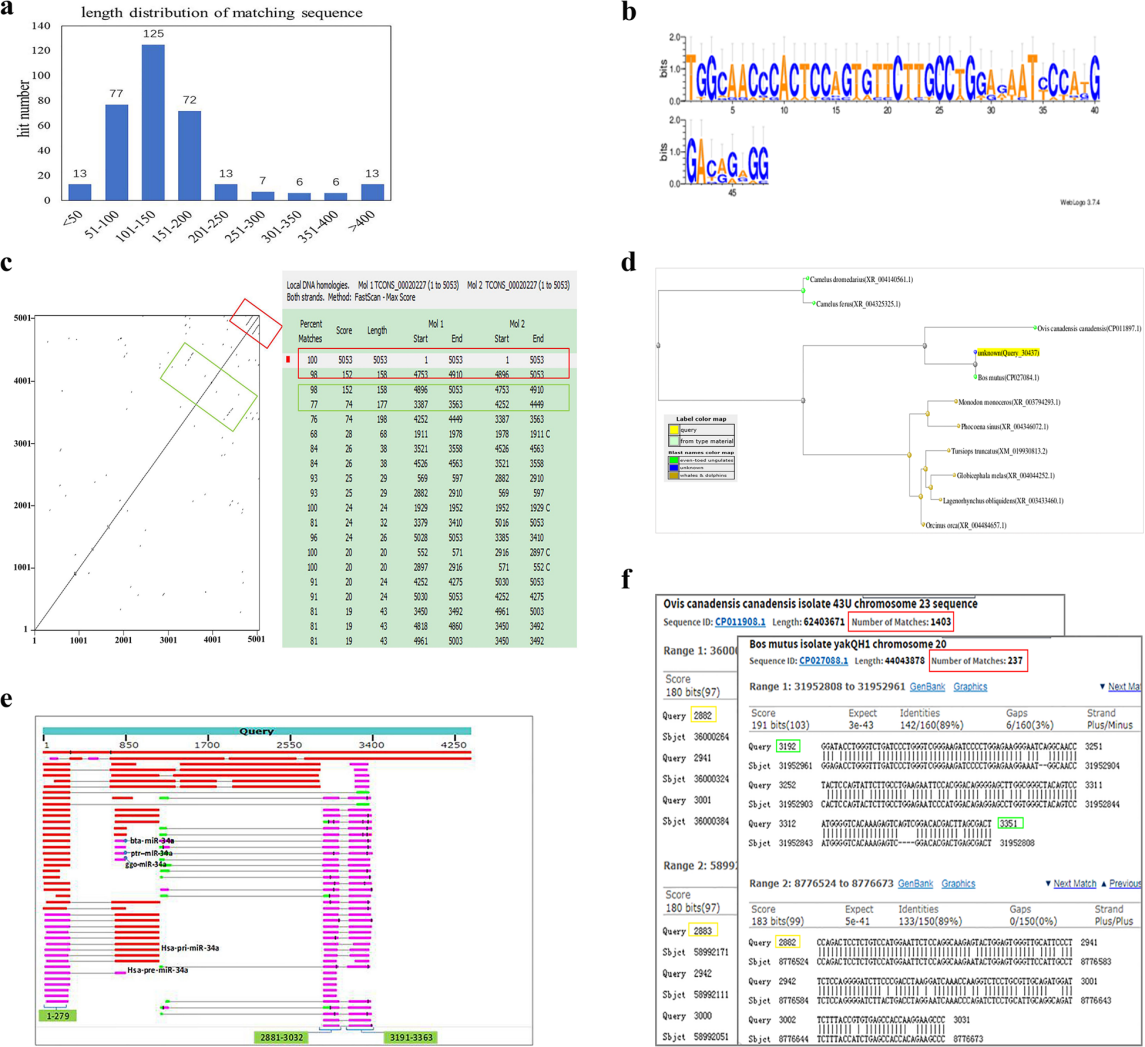
Analysis of conservation properties of the lncRNA alignments between yak and cashmere goat. **a** Length distribution of the matching sequence of all the hits by NCBI BLAST-2.9.0+. **b** Sequence logo of the highly conserved sequences between TCONS_00008989 and its aligned 10 cashmere goat lncRNAs using WebLogo3. **c** Dot plot of TCONS_00020227 and the detailed ranges of the repeats on TCONS_00020227 were analyzed using dottup website and Clone Manager software, respectively. The boxes with the same color indicate the same region of TCONS_00020227, and the red and green boxes represented two pairs of repeats on TCONS_00020227. **d** The Blast Tree View of TCONS_00016111 produced using BLAST pairwise alignments with neighbor joining algorithm. Query_55251 presented TCONS_00016111, and other terms on the tree were the first seven sequences of TCONS_00016111 BLAST result. **e** Graphic summary of selected 100 sequences in defaulted first page of TCONS_00016111 BLAST result. Three matching regions of TCONS_00016111 are marked in green box. **f** Typical alignments example of the two key matching regions, about 2881–3029 and 3191–3365, in the chromosome of *Ovis canadensis canadensis* and *Bos mutus* (yak). The number of matches in each of these two chromosomes is highlighted using red box

In the above section, the sequence conservation of the 110 DELs in yak was investigated by compared with cashmere goat lncRNA data during HFs cycle using NCBI BLAST-2.9.0+. Then, a detailed analysis of the sequence conservation properties of these aligned lncRNAs were performed. In the result of NCBI BLAST-2.9.0+, total of 287 alignments including 332 hits were obtained in goat lncRNA data, which were aligned by 24 DELs of the yak database (Additional file 6) [19]. Length of the matching regions (identities) of the hits was distributed between 36 and 3765 bp, and the matching percentage was more than 80% (Additional file 6). Figure 4a shows the length distribution of the matching sequence of all the hits by BLAST-2.9.0+, which indicates that the number of the matching sequence distributed between 101 and 150 bp is the most, and the matching regions distributed between 51 and 200 accounting for 82% of all the hits [19] (Fig. 4a, Additional file 5). Interestingly, the analysis of every aligned lncRNA found that most of the queried lncRNAs of the yak had numerous alignments with cashmere goat lncRNA dataset while the matching regions on one lncRNA in the yak were constrained (Additional files 6 and 7). Table 1 presents partial queries that aligned more than 10 lncRNA sequences in cashmere goat dataset. For example, the matching region of lncRNA TCONS_00027937 with a high sequence conservation that aligned to 35 lncRNA sequences of the cashmere goat dataset while all of these alignments roughly matched with the region of 43–160 of TCONS_00027937, the region might be an important element for the function of TCONS_00027937. Then, the lncRNAs TCONS_00008989 and TCONS_00020227 were randomly selected, along with lncRNA TCONS_00016111, which had the longest matching sequence to 3765 bp, were used to perform further detailed analysis for their matching regions.

**Table 1:** Information of DELs aligned with numerous lncRNA sequences of cashmere goat

Query	Length	Number of matched alignments	Partner_gene	Regions of matching sequence
TCONS_00008989	1549	13	GPRC5D	243–442
TCONS_00020227	5053	31	TMEM231	2506–3121,4251-4452,4723-4898,4886–5053
TCONS_00058749	2278	25	ASAH2	172–359, 1673–1863
TCONS_00055139	2913	12	BACH1	920–1463
TCONS_00033093	2364	11	EMX2	1929–2091
TCONS_00018581	229	14	FER	1–229
TCONS_00016111	4400	25	GPR157	690–4400(2),103–279(2),277–689(1),2881–3029,3191–3365
TCONS_00027937	6622	35	ISPD	43–160
TCONS_00000176	839	16	LIPG	1–133,131–267
TCONS_00016755	1962	15	LOC102274179	923–1962
TCONS_00027432	1635	30	OAF	581–1397(3),1058–1400,1–171
TCONS_00031781	223	15	COLEC12	1–223
TCONS_00058073	3188	11	LOC102267365	2867–3059

Note: The number in parentheses refer to the aligned numbers in that region (relative less), and the remaining aligned numbers are assigned to other matching regions

For TCONS_00008989, all the alignments of cashmere goat data were roughly mapped to the region of 243–442 of TCONS_00008989. Then, a local multiple sequence alignment was manually performed in the matching region of TCONS_00008989 with other aligned 11 different sequences in cashmere goat. The result showed that the matching region between 315 and 356 of TCONS_00008989 about 40 bp with high sequence identity among 10 of the alignments (Figure S2). The sequence logo of this highly identical region among alignments was presented using WebLogo3 (http://weblogo.threeplusone.com/) (Fig. 4b). This short sequence might be an essential binding module for dictating the function of TCONS_00008989. Besides, it was speculated that a common feature of sequence conservation of lncRNAs was a short highly conserved sequence shared by multiple lncRNAs of one species or various species.

TCONS_00020227 is an intronic lncRNA, which was aligned to 31 lncRNA sequences of cashmere goat data, mainly matched with 4 regions: 2506–3121, 4251–4452, 4723–4898, and 4886–5053 within TCONS_00020227 (Additional file 6, Table 1). Compared with TCONS_00008989, the matching regions in TCONS_00020227 were relatively varied. The presence of repeats on TCONS_00020227 was analyzed using the Dot Plot of TCONS_00020227 on the dottup website. Two pairs of repeats appeared on TCONS_00020227 (Fig. 4c). The detailed regions of the repeats were presented by pair-wise alignment using Clone Manager software (Fig. 4c), finding that the latter repeats were coincident with the matching regions of 4723–4898 and 4886–5053, detected by NCBI BLAST-2.9.0+ (Fig. 4c and Table 1). The slight difference in the sequence value might be due to the use of distinct analysis methods, but the ranges were comparable. Another repeats appearing in the Dot Plot of TCONS_00020227 were also observed using Clone Manager:4252–4449 (equivalent to 4251–4452 detected by NCBI BLAST-2.9.0+), which repeated with the range of 3387–3563, despite a lower match percentage. This result showed that the matching regions repeatedly existed in TCONS_00020227 and further indicated the importance of the matching sequences. Besides, the data also indicated the effectivity of the BLAST+ result.

Finally, the sequence conservation of TCONS_00016111 was analyzed. As TCONS_00016111 had a longer matching region to 3765 bp, the sequence conservation of TCONS_00016111 was explored using Nucleotide BLAST in nucleotide collection databases with default parameters. The BLAST result showed that lncRNA TCONS_00016111 mapped to multiple species genomes, including *Ovis canadensis canadensis*, whales, *Bos taurus*, *Capra hircus*. Most of these alignments were ncRNAs (Figure S3). Interestingly, the distance tree of the selected alignments of the top 15 organisms in the defaulted first page of BLAST result showed that TCONS_00016111 with *Ovis canadensis canadensis* and whales were closer than bovines at the distance tree (Figs. 4d and S3), suggesting a relationship between *Ovis canadensis canadensis*, whales, and yaks in terms of some evolutionary traits. Moreover, two matching regions 2881–3029 and 3191–3365 of TCONS_0001611 searched using NCBI BLAST-2.9.0+ between yak and cashmere goat lncRNA data emerged in nearly all of the mapped sequences in the first page using Nucleotide BLAST (Fig. 4e). More importantly, these two regions were largely repeated in the genomes of *Ovis canadensis Canadensis* (bighorn sheep) and *Bos mutus* (yak) in different chromosomes, the number of repeats was from hundreds to more than one thousand. Figure 4f shows an example of the searched results. These two regions simultaneously emerged in the genome in abundance, and were found to be partly reverse-complementary so that the region 2881–3365 could form a stable secondary structure (Figure S4), which might be one of the conserved secondary structures of TCONS_0001611. This finding was consistent with the reported conservation properties of lncRNAs in terms of the conserved secondary structure [13]. In addition, miR-34a in cattle, *Pan troglodytes*, and *Gorilla gorilla*, and the pre-miR-34a and pri-miR-34a in human were found within the alignments of TCONS_00016111 (Fig. 4e), implicating that TCONS_00016111 also act as a miRNA precursor.

## Discussion

The development of HFs cycle is the characteristic of hair growth in mammals, including anagen, catagen, and telogen [57]. The transition between different phases of this cycle is controlled by the unique regenerating system of follicular epithelial and mesenchymal cells with the interaction of their multiple molecular signals [58]. LncRNAs, as key regulatory molecules have been found to play an important role in the HFs cycle. They have been extensively explored in fur-producing animals due to the economic benefit of animal fibers and observable changes in hair growth phenotype [17, 59, 60]. The hair growth pattern of yak is similar to that of cashmere goat, presenting a seasonal phenomenon under the control of photoperiod. This study first investigated the expression profile of lncRNAs during the HFs cycle and analyzed the potential function of DELs on yak. In the HFs cycle, anagen is the longest stage occupying more than half of the whole cycle (1 year) [50]. Division of the HFs cycle in yak has been rarely reported to data. In this study, samples in five time points of yak were employed to perform lncRNA sequencing, including Jan., Mar., Jun., Aug., and Oct.. After a preliminary analysis of the DELs data between the comparison groups, Oct. was selected as the optimum time in the anagen with Jan. (catagen) and Mar. (telogen) for further functional analysis. The samples in the HFs cycle of yak were collected referring to the phase division of a cashmere goat data and based on the phenotypic changes in yak [50]. Previous studies reported that the phase division of the HFs cycle in Shaibei cashmere goat in North China was as follows: the anagen was from Apr. to Oct., the catagen was from Nov. to Jan., and the telogen was from Feb. to Mar.. In this study, DELs between Jan., Mar. and Oct. in the sequencing date were relatively more than those in other comparison groups, and the distinct phases of HFs cycle could be significantly distinguished (Fig. 1c). Jun. might be a transition phase from the telogen to anagen. Oct. had more changes in the anagen phase compared with Jun. and Aug., which was consistent with a previous study reporting that the ratio of secondary to primary follicles (S/P) in Oct. was the highest in cashmere goat [50]. After confirming the optimum three phases of the HFs cycle in the yak, the functional analysis of the DELs in the three distinct phases were performed.

LncRNAs could affect the gene expression in the immediate genomic vicinity (in cis) [12, 14]. In the present study, the nearest gene within 100 kb of lncRNAs was searched and used to predict the potential function of DELs by GO and KEGG enrichment analysis. The result showed that in the transition from the anagen to catagen (Oct.-vs-Jan.), the primarily enriched GO terms of cell communication and metabolism (e.g. cell surface receptor signaling, carbohydrate metabolic process, and protein O-linked mannosylation) were down-regulated, and cell transformation increased (e.g. keratinization and mitotic cell cycle). In the transition from the catagen to the telogen (Jan.-vs-Mar.), the function of nutrition metabolism (e.g. thiamine transport, response to glucose, and regulation of insulin secretion) was mainly enriched, which might be related to the preparation for follicle reset. Interestingly, the functions associated with protein ubiquitination was enriched several times in the transition from the telogen to the anagen (Mar.-vs-Oct.). Previous studies reported that the ubiquitin-mediated proteolysis pathway was important for distinguishing the SFs from PFs of cashmere goats [46, 47], which coincided with the present data that Oct. might be the phase with thriving SFs and the growth of PFs might be vigorous in Mar. [50]. The signaling pathways involved in HFs morphogenesis were important for maintaining the normal periodical cycle of hair growth [5]. KEGG enrichment analysis found that Wnt signaling, which was widely reported playing a curial role in HFs development [48, 61], was enriched in the Jan.-vs-Mar. group. The VEGF signaling pathway was enriched in both the Jan.-vs-Mar. and Mar.-vs-Oct. groups. VEGF was involved in the regulation of the HFs cycle by inducing the angiogenesis of dermal papilla [9]. The HIF-1 signaling pathway was enriched in the Mar.-vs-Oct. group, which might be associated with the hypoxia adaptability of yak [62]. From a holistic perspective, the pathways that emerged in the distinct phases seemed to orchestrate a dynamic genesis of the HFs cycle at a molecular level that corresponded with the biological process of HFs development.

A large number of DELs during the HFs cycle in various species were examined following the rapid development of genome sequencing technology, but the identified functional lncRNAs were rare, partly due to the lower level of sequence conservation of lncRNAs across species [12]. The DELs with nearby paired DEGs were screened and the sequence conversation of these lncRNAs between the yak and cashmere goat during HFs cycle was further analyzed to obtain more reliable DELs affecting the HFs growth. Twenty-four DELs were found with sequence conservation between the yak and the cashmere goat lncRNA data to different degrees. Sequence conversation was an important factor in revealing the function of lncRNAs. The well-studied lncRNAs, such as Xist, HOTAIR, and MALAT1, were reported with higher sequence conversation or conserved domain [25–27], and it was identified that a highly conserved sequence element of lncRNA was essential for its function [63], although a lack of sequence conservation did not directly imply a lack of function [64]. Previous studies reported that lncRNAs were specifically expressed in tissue and species with poor conservation at the sequence level [11, 65]. In the present study, yak and cashmere goat all belong to the Bovidae family of ruminants. Also, the seasonal cyclic development of HFs in yaks was similar to that in cashmere goats, including anagen, catagen, and telogen, providing a well molding to study the sequence conservation of lncRNAs for the same trait in different species. The NCBI BLAST-2.9.0+ result showed that 24 lncRNAs among 110 DELs of the yak were aligned with cashmere goat lncRNA dataset, accounting for 20% of total queries (Additional file 6). Many well-studied lncRNAs exhibit well-conserved RNA secondary structures [13], or the highly conserved region of a lncRNA contains a functional motif that regulates one biological process [66]. The sequence conservation properties of these aligned lncRNAs were analyzed in detail. A large number of the queried alignments in yak were aligned to multiple lncRNA sequences in cashmere goat dataset, while the matching regions in the queried lncRNAs of yak were constrained; these matching sequences probably contained the elements essential for their activity [67–69]. In the present study, three lncRNAs were selected for further detailed analysis. Among these lncRNAs, TCONS_00008989 aligned to 13 lncRNA sequences of cashmere goat data. All of these alignments nearly matched with one region: about 200 bp of TCONS_00008989, and approximately 40 bp was highly conversed, which might be a key recognition sequence of TCONS_00008989. Another 2 selected lncRNAs TCONS_00020227 and TCONS_00016111 were respectively aligned to 25 and 30 lncRNA sequences in cashmere goat dataset. The matching sequences of TCONS_00020227 were repeatedly observed in TCONS_00020227, which might be a commonality of many sequence conserved lncRNAs. TCONS_00016111 was matched with a longer sequence of a cashmere goat lncRNA. Hence, Online BLAST was performed, revealing that the molecular evolution tree of TCONS_00016111 was different from the species tree of yak (http://asia.ensembl.org/info/about/speciestree.html). The distance of yak in this molecular evolution tree was closer to *Ovis canadensis canadensis* and whales instead of cattle in species tree (Figs. 4d, S3). It was well established that whales were more closely related to cows and their relatives compared with other mammal such as donkeys and horses and their perissodactyl relatives [70]. In addition, the oldest fossil of an approximately 50-million-year-old whale was discovered in the coeval sediments of Indo-Pakistan [71, 72]. This area was close to the habitat of wild yaks on the Tibetan plateau, which also provided a clue for the hypothesis of an evolutionary relationship between whales and yaks. A transition of lncRNAs was reported from conserved lncRNAs toward an increasing number of lineage- and organ- specific lncRNAs during their development [11]. On the other hand, lncRNAs were found to be ancient components in the evolution of vertebrate genome [29], it was believed that a few highly conserved lncRNAs in evolution still existed; for example, the lncRNA TCONS_00016111 found in the BLAST analysis in the present study. Further, notably, two matching regions that could form a stable secondary structure in TCONS_00016111 were largely repeated from hundreds to more than one thousand in the genome of *Ovis canadensis canadensis* and yak (Figures S4 and 4f), implicating that the repeatedly emerging sequences might act as an important module to recognize RNA, DNA, or protein for the activity on their genomic loci [13, 63]. Meanwhile, these data also indicated the efficiency of the aligned conservation elements using NCBI BLAST-2.9.0 + .

## Conclusions

This study was novel in presenting the data on the lncRNAs of yak during the HFs cycle using lncRNA-seq. The functions of the identified DELs were indirectly predicted via the enrichment analysis of the function of their nearest mRNAs. In addition, several sequence-conserved lncRNAs between yak and cashmere goat in the HFs cycle were searched using NCBI BLAST-2.9.0+, and the sequence conservation properties were analyzed. The conserved sequence elements might play an important role in the regulation of the HFs cycle. The finding of the present study might provide valuable insight into the study of lncRNAs functionality and mechanism in the HFs cycle.

## Supplementary information

**Additional file 1.**

**Additional file 2.**

**Additional file 3.**

**Additional file 4.**

**Additional file 5.**

**Additional file 6.**

**Additional file 7.**

**Additional file 8.**

**Additional file 9.**

**Additional file 10.**

**Additional file 11.**

**Additional file 12.**

## Data Availability

The datasets generated and analysed during the current study are available in the NCBI repository, accession number: PRJNA550233 (https://www.ncbi.nlm.nih.gov/bioproject/PRJNA550233). The data supporting the conclusions of this study are available within the additional files.

## Abbreviations

DEGs: Differentially expressed genes
DELs: Differentially expressed lncRNAs
HFs: Hair follicles
KEGG: Kyoto Encyclopedia of Genes and Genomes
lncRNAs: Long non-coding RNAs
