# Supplementary figures and images for: Genome-wide detection and sequence conservation analysis of long non-coding RNA during hair follicle cycle of yak
Source: BMC Genomics. 2020 Oct 1;21:681. doi: 10.1186/s12864-020-07082-z (PMC7528256; doi:10.1186/s12864-020-07082-z)

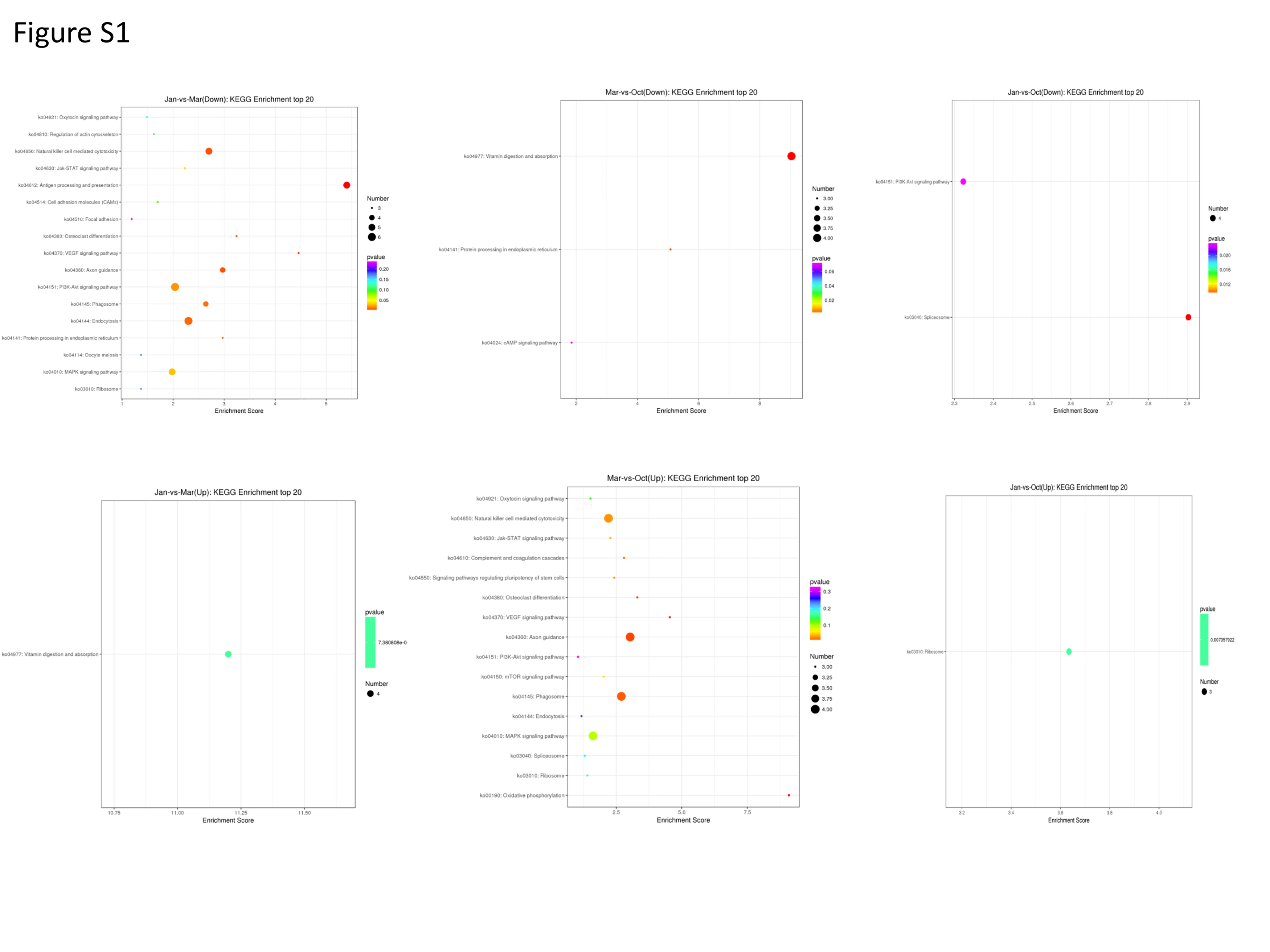

Supplement: Supplementary file 9 — Additional file 9. [file 12864_2020_7082_MOESM9_ESM.tif]

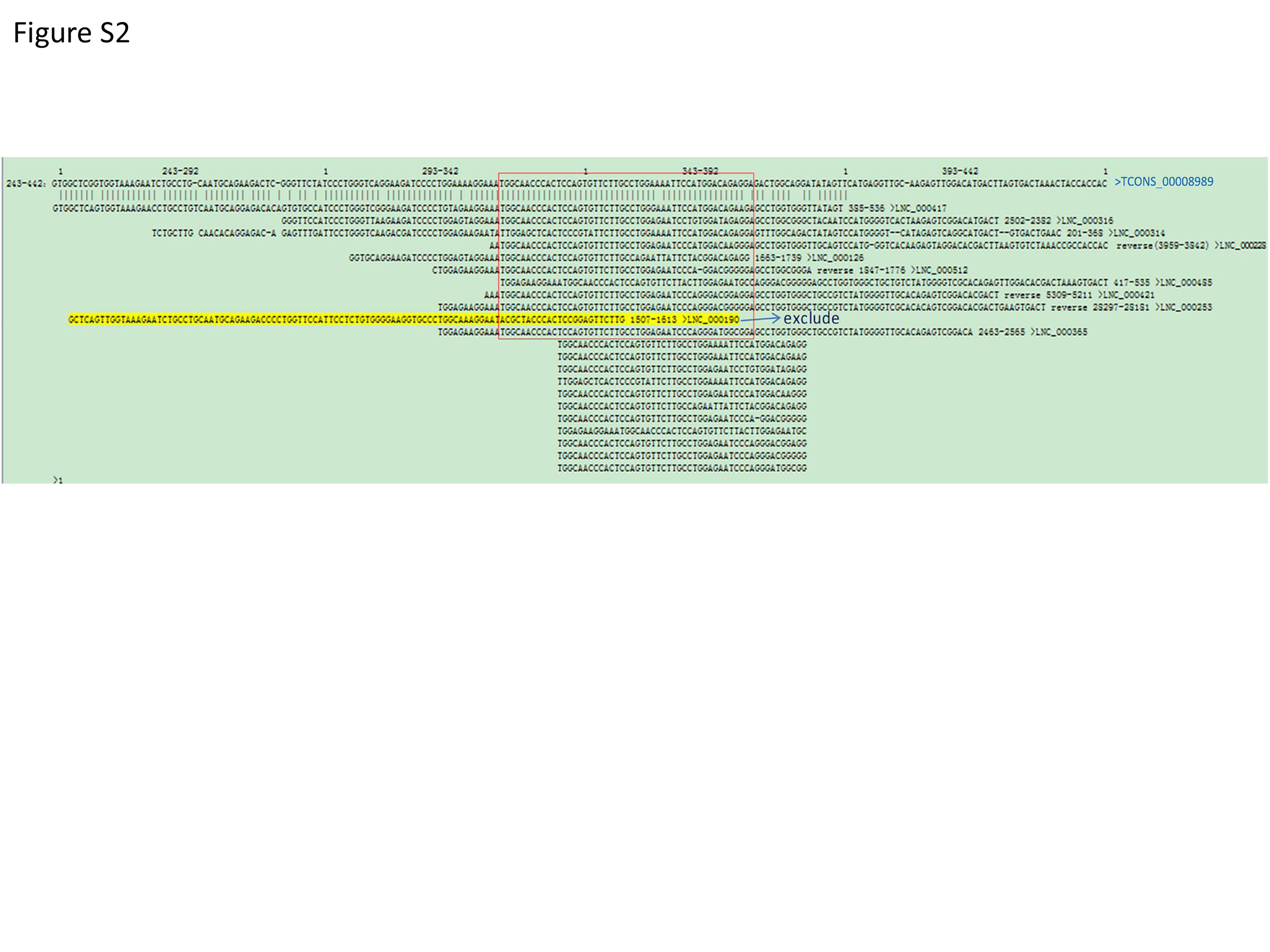

Supplement: Supplementary file 10 — Additional file 10. [file 12864_2020_7082_MOESM10_ESM.tif]

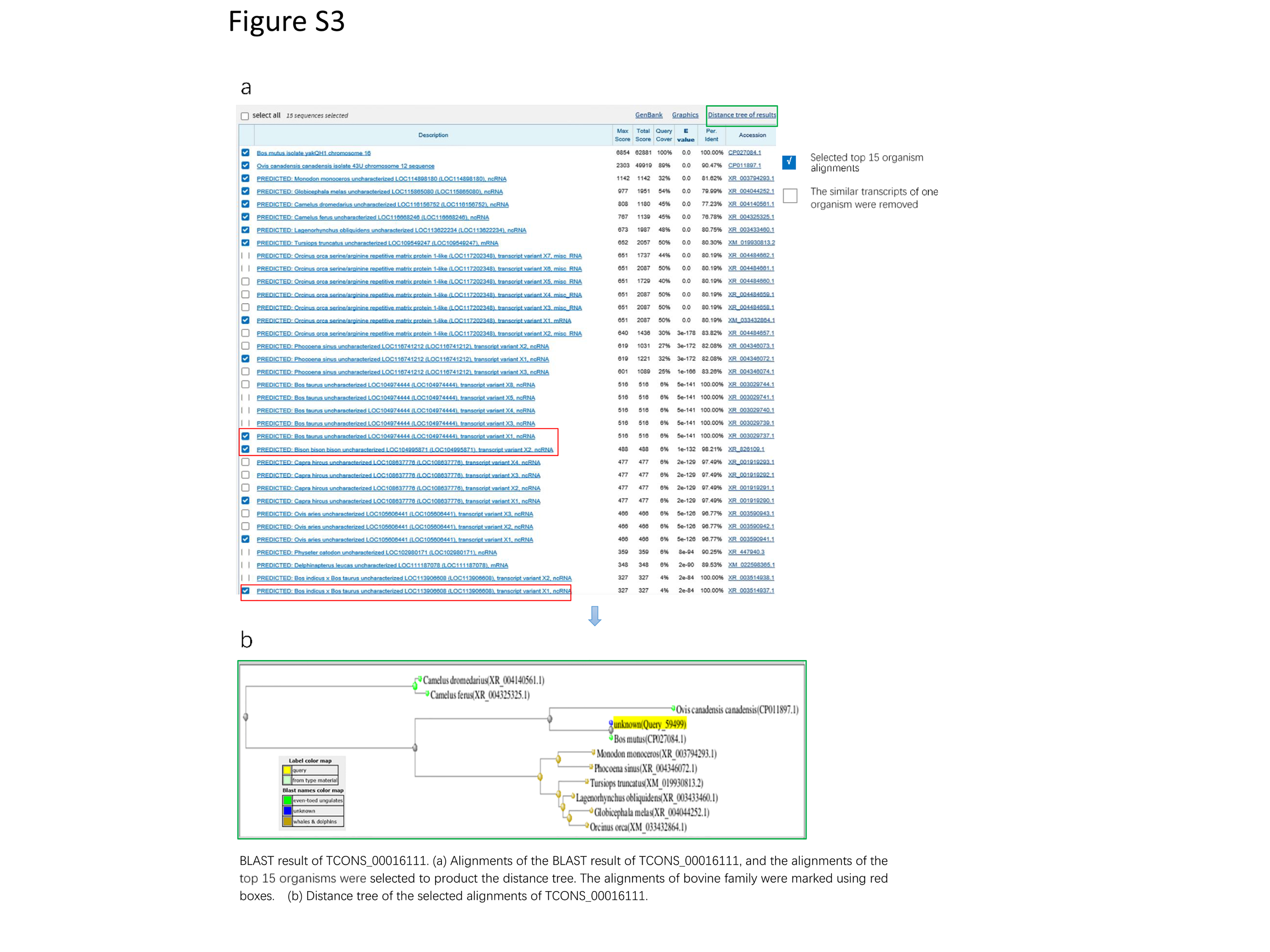

Supplement: Supplementary file 11 — Additional file 11. [file 12864_2020_7082_MOESM11_ESM.tif]

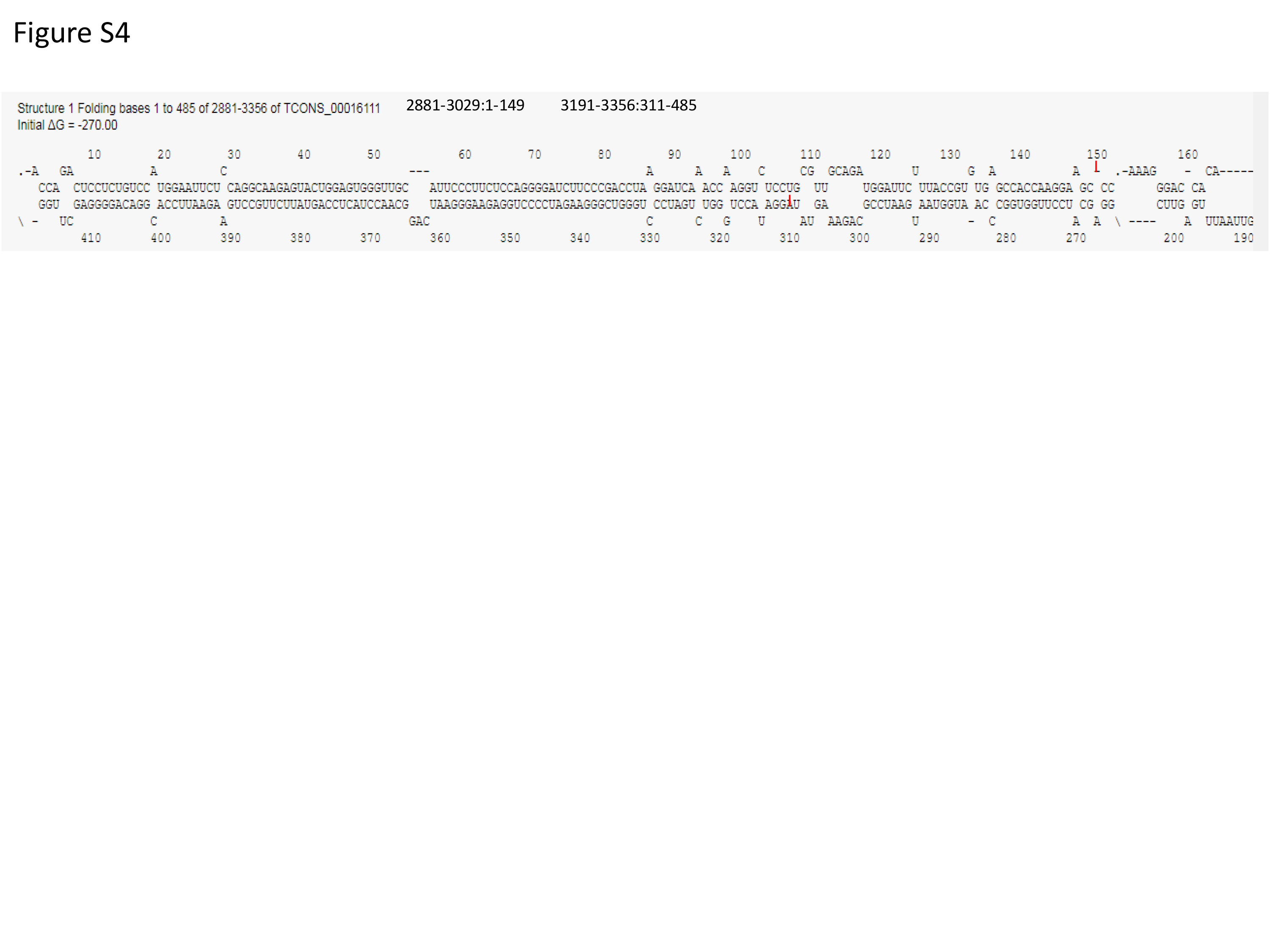

Supplement: Supplementary file 12 — Additional file 12. [file 12864_2020_7082_MOESM12_ESM.tif]
